# Intra-Anal Imiquimod Cream against Human Papillomavirus Infection in Men Who Have Sex with Men Living with HIV: A Single-Arm, Open-Label Pilot Study

**DOI:** 10.3390/jcm10194477

**Published:** 2021-09-28

**Authors:** Duygu Durukan, Tiffany R. Phillips, Gerald L. Murray, Jason J. Ong, Andrew E. Grulich, I. Mary Poynten, Fengyi Jin, Catriona S. Bradshaw, Ivette Aguirre, Julie Silvers, Helen Kent, Steph Atchison, Prisha Balgovind, Alyssa Cornall, Marcus Y. Chen, Christopher K. Fairley, Eric P. F. Chow

**Affiliations:** 1 Central Clinical School, Monash University, Melbourne, VIC 3004, Australia; tiffany.phillips@monash.edu (T.R.P.); Jason.Ong@monash.edu (J.J.O.); catriona.brashaw@monash.edu (C.S.B.); mchen@mshc.org.au (M.Y.C.); cfairley@mshc.org.au (C.K.F.); 2 Melbourne Sexual Health Centre, Alfred Health, Carlton, VIC 3053, Australia; I.Aguirre@alfred.org.au (I.A.); JSilvers@mshc.org.au (J.S.); kents@bigpond.net.au (H.K.); 3 Murdoch Children’s Research Institute, Parkville, VIC 3052, Australia; gerald.murray@mcri.edu.au (G.L.M.); Steph.Atchison-Wright@rch.org.au (S.A.); prisha.balgovind@mcri.edu.au (P.B.); alyssa.cornall@mcri.edu.au (A.C.); 4 Centre for Women’s Infectious Diseases, The Royal Women’s Hospital, Parkville, VIC 3052, Australia; 5 Department of Obstetrics and Gynaecology, The University of Melbourne, Parkville, VIC 3052, Australia; 6 The Kirby Institute, UNSW Sydney, Sydney, NSW 2052, Australia; agrulich@kirby.unsw.edu.au (A.E.G.); mpoynten@kirby.unsw.edu.au (I.M.P.); jjin@kirby.unsw.edu.au (F.J.); 7 Melbourne School of Population and Global Health, The University of Melbourne, Melbourne, VIC 3052, Australia

**Keywords:** prevention, anal cancer, intervention, HPV, men, anus

## Abstract

Men who have sex with men (MSM) living with HIV have a high prevalence and incidence of anal high-risk human papillomavirus (hrHPV) and anal cancer. We conducted an open-label, single-arm pilot study to examine the tolerability of imiquimod cream among MSM aged ≥18 years, living with HIV, who tested positive for anal hrHPV at Melbourne Sexual Health Centre between April 2018 and June 2020. We instructed men to apply 6.25 mg imiquimod intra-anally and peri-anally 3 doses per week for 16 weeks (period 1) and then one dose per week for a further 48 weeks (period 2). Twenty-seven MSM enrolled in period 1 and 24 (86%) applied at least 50% of doses. All men reported adverse events (AEs), including 39.5% grade 1, 39.5% grade 2, and 21% grade 3 AEs on at least one occasion. Eighteen MSM (67%) temporarily stopped using imiquimod during period 1, most commonly due to local AEs (*n* = 11) such as irritation and itching. Eighteen MSM continued in period 2 and all applied at least 50% of doses with no treatment-limiting AEs reported. Imiquimod 3 doses per week caused local AEs in most men and was not well tolerated. In contrast, once-a-week application was well tolerated over 48-weeks with no treatment-limiting AEs.

## 1. Introduction

Human papillomaviruses (HPV) are the most common sexually transmitted infection globally [1]. The majority of anogenital squamous cell cancers (ASCC) are caused by a persistent infection with the high-risk HPV (hrHPV) types 16 and 18 [2], including 85–95% of anal cancers [3]. Men who have sex with men (MSM) living with HIV have a high incidence of ASCC that is estimated to be 85 cases per 100,000 person-years, which is over four times higher than MSM without HIV (19 per 100,000 person-years) and 40 times higher than the general population (2.0 per 100,000 person-years) [4,5]. The highest risk group is MSM living with HIV who are over 60 years of age, with incidence rates reaching 107.5 cases per 100,000 person-years [4,5]. The prevalence of anal hrHPV infection in this group is correspondingly high [6,7,8]. MSM living with HIV may also have a faster progression from low-grade lesions caused by hrHPV to higher grade lesions compared with HIV-negative populations [9,10].

The incidence of ASCC in MSM living with HIV has been on the rise [11,12,13,14,15,16]. This has been attributed to an increasing life expectancy among people living with HIV due to the availability of antiretroviral therapies and consequent prolonged anal hrHPV infection [17,18,19]. HPV16 causes 85% of ASCC in all MSM, and 65% in MSM living with HIV [20]. Screening for hrHPV-induced lesions such as anal intraepithelial neoplasia has been recommended by some health services [21,22]. However, this is controversial due to the invasive nature of treatments and the high recurrence rates following treatment [23,24,25] Vaccination is highly likely to be an effective biomedical prevention strategy in MSM under 26; however, its efficacy against anal cancer has not yet been proven [26,27].

Most HPV infections are cleared spontaneously by the innate immune system [28]. Imiquimod is an immune response modifier that works by stimulating both innate and adaptive immunity, as well as inducing apoptosis. It is effective against anogenital warts caused by low-risk HPV genotypes 6/11 [29] but has not been evaluated for other HPV types. As imiquimod stimulates the immune system in the application area, local adverse events are common and may include burning, itching, and pain at the application site due to mucocutaneous inflammation [5] Due to the sensitive nature of anal and perianal areas, we designed this pilot clinical trial to test the tolerability of intra-anal imiquimod at the dose which has been successful against anogenital warts caused by low-risk HPV genotypes.

Our primary aim was to determine the tolerability of intra-anal imiquimod used 3 times per week for 16 weeks in MSM living with HIV followed by a maintenance dose of once per week for 48 weeks.

## 2. Materials and Methods

### 2.1. Study Setting and Participants

The CHAMP (Clearing high-risk human papillomaviruses among men who have sex with men pilot) study was a 64-week, single-arm, open-label, pilot clinical trial conducted at Melbourne Sexual Health Centre, the largest urban public sexual health centre in Victoria, Australia. Eligibility criteria were any MSM living with HIV who was older than 18 years of age at recruitment. Patients diagnosed with anal cancer in the preceding 12 months or those living with an autoimmune disease were not eligible as imiquimod has the potential for systemic immunostimulant activity [30].

For enrolment, the study team screened our clinic’s daily patient lists and marked eligible patients as potential candidates to be approached by their treating clinician. A member of the study team explained the study details to the interested patients and obtained written consent for an anal swab to test for hrHPV. Men who tested positive for anal hrHPV were invited to return to the clinic. Men who tested negative for hrHPV were notified of their results over text message and no further follow-up was done.

The study was conducted in two periods: period 1, an intervention period which consisted of 3 doses per week application for 16 weeks, followed by period 2; a maintenance period of one dose per week application for a further 48 weeks. A three-times-per-week regimen was based on the recommended protocol for imiquimod use to treat anogenital warts [29]. However, as the cream was to be inserted into the anal canal, we recommended using half a sachet (half the dose used for anogenital warts) to reduce the likelihood of systemic absorption through the anal mucosa [23]). This dose has also been used in a clinical trial examining imiquimod use for the treatment of anal intraepithelial neoplasia and we wanted our results to be consistent with this study [23]. Participants were scheduled for a clinic appointment at week 16 of period 1 and at 24 and 48 weeks of period 2. Enrolled participants received 24 sachets of 5% imiquimod cream, containing 12.5 mg of imiquimod per sachet to be used in period 1. We instructed men to apply half a sachet (6.25 mg) three times per week intra-anally and peri-anally on non-consecutive days before bedtime, avoid having receptive anal intercourse for 4–6 h and wash the area the following morning. We instructed period 2 participants to apply half a sachet of cream intra-anally once per week (6.25 mg) for an additional 48 weeks. Imiquimod was provided in packs of 13 sachets at each period 2 visit. We provided all participants with an imiquimod application instruction sheet (Supplementary Figure S1).

The quadrivalent HPV vaccine (Gardasil, Merck & Co., Whitehouse Station, NJ, USA) was offered to enrolled participants with hrHPV to prevent new infections and was given following the recommended dose and schedule at 0, 2 and 6 months. The study was approved by the Alfred Hospital Ethics Committee, Melbourne, Australia (473/17). The study was conducted in compliance with the regulatory requirements, good clinical practice (GCP) and the ethical principles of the Declaration of Helsinki as adopted by the World Medical Association and reported as per the CONSORT 2010 Guidelines. This trial was registered at the Australian New Zealand Clinical Trials Registry (ACTRN12617001355369) on 27 September 2017.

### 2.2. Laboratory Methods

Each participant provided two anal swabs in period 1 (Baseline and week 16) and two in period 2 (week 24 and week 48). To obtain the specimens, clinicians removed a Copan flocked swab from its tube, moistened with sterile sodium chloride, wiped it around the perianal area and then inserted the swab into the anal canal 2–3 cms, rotated and removed the swab. The swab was then immersed and vigorously agitated in a ThinPrep sample vial filled with 20 mL of PreservCyt^®^ Solution (Hologic Inc., Marlborough, MA, USA) and discarded. The ThinPrep sample vial was capped, labelled, and sent to the laboratory for testing. Screening samples were tested in real-time using the Roche Cobas 4800 HPV test (Roche, Branchburg, NJ, USA) which detects 14 h HPV strains in a pooled result [31]. Samples from week 16 (period 1), week 24 (period 2) and 48 (period 2) were stored at −80 °C. One millilitre of samples was pelleted by centrifugation, resuspended in 200 mL PBS, then extracted on the MagNA Pure 96 platform using the DNA and Viral NA Small Volume Kit (Roche), and batch tested according to manufacturer’s instructions using Seegene Anyplex II HPV HR detection multiplex assay (Seegene Inc., Seoul, Korea) [32].

### 2.3. Participant Follow-Up

Follow-up was conducted via SMS, clinic visits, and self-filled questionnaires (Table 1). Participants completed three questionnaires at each follow-up point; (i) the previously validated EQ-5D-5L to measure health-related quality of life, (ii) a sexual functioning questionnaire adapted from the previously validated International Index of Erectile Function, and (iii) a tolerability questionnaire regarding anal symptoms [33,34,35]. The tolerability questionnaire asked men about the presence and the severity of local adverse events (redness, swelling, itch, irritation, dryness, burning, pain, tenderness, flaking, peeling of the skin, exudation from skin) as well as systemic symptoms (fatigue and headache). The questionnaire asked participants to grade the severity of the symptoms (mild, moderate, severe) which were analysed as grade 1, grade 2 and grade 3, respectively; the frequency (every day, few times a week, few times a month, once a month) and if they wanted to interrupt treatment because of the AE. Participants who reported non-adherence to the prescribed dosing or reported AEs were contacted via phone for a review of symptoms. Experienced sexual health doctors (CKF, CSB, MYC) determined whether the patients should have a period of interruption in case of grade 2 and above AEs. Participants were asked to stop using imiquimod for four weeks and re-commence at a reduced dose of once per week for a further four weeks. Men who were adherent with follow-up during period 1 were invited to continue in period 2.

### 2.4. Study Outcomes and Statistical Analysis

The primary outcomes of the study were the estimates and the associated confidence intervals (CI) of HPV positivity and tolerability. We defined “tolerable” as completing at least 50% of scheduled doses during each period. “Intolerable” was defined as tolerating less than 50% of doses. We anticipated if 20% of participants could not tolerate the full treatment for 16 weeks then the 95% CI around this estimate would be 8–39% with 30 enrolled participants. With an estimated hrHPV prevalence of 55–60% among MSM living with HIV at our clinic [7], we anticipated that 70 MSM would need to agree to initial HPV testing. We used descriptive statistics to summarise the main outcomes of the study and Kaplan–Meier plots to illustrate survival until Grade 1 and Grade 2–3 AEs. A Chi-squared test with Yates’ correction was used for qualitative data. The level of significance was chosen to be two-sided *p* < 0.05.

EQ-5D-5L responses were converted to utility index scores using the UK general population-based algorithm as no value sets have been made for Australia at the time of reporting [36,37]. The Wilcoxon sign rank test was used to identify changes in utility scores from baseline visit to subsequent visits. All analyses were conducted in Stata (version 16 Stata Corp., College Station, TX, USA).

## 3. Results

Participants were recruited between April 2018 and December 2018 and follow-up was completed in June 2020. We approached 110 men, 72 agreed to screening, 43 had confirmed hrHPV (59.7%, 95%CI 48.2–70.3) and 30 agreed to enrolment in period 1. The median age was 50 (IQR 40–58). All patients were taking combination antiretroviral therapy. Most men self-reported being unvaccinated against HPV (*n* = 26/28, 92.9%, 95%CI 77.4–98.0). The last follow-up sample was obtained in July 2020.

### 3.1. Tolerability of Imiquimod

#### 3.1.1. Period 1

**Figure 1:**
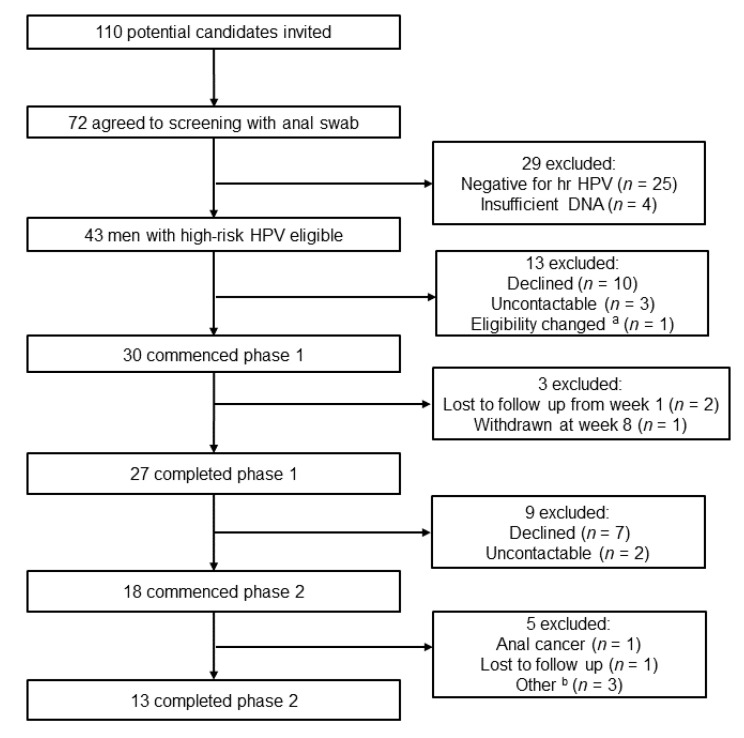
Flowchart of participant progress through each period of the trial. ^a^ One participant had an active herpes genitalis episode and had to be excluded from the study. ^b^ Other reasons were moving interstate or overseas (*n* = 2) and work commitments (*n* = 1).

**Figure 2:**
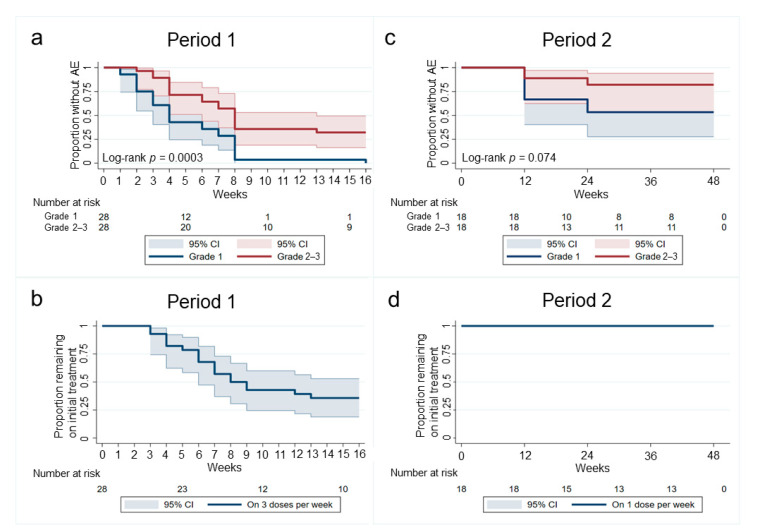
Kaplan–Meier Survival curves demonstrating time to adverse events in period 1 and period 2. (**a**,**c**) demonstrate the proportion of men in period 1 and period 2 who remained without adverse events over the corresponding study periods, as well as those who developed grade 1 (blue line) and grade 2 or 3 adverse events (red line). (**b**,**d**) demonstrate the proportion of men who remained on the initially prescribed imiquimod treatment in period 1 (3 doses per week) and period 2 (1 dose per week).

Thirty MSM agreed to commence Period 1 and 27 completed the week 16 follow-up visit (Figure 1). Two men did not commence treatment or respond to follow-up, and one man withdrew from the study at week 8. Overall, 85.7% of men took at least 50% of imiquimod doses during period 1 (IQR 68.5–94.3). Median number of doses taken over 16 weeks was 32 (IQR 27–43). Kaplan–Meier analysis demonstrated that all men experienced a grade 1 AE and most reported a grade 2–3 AE during period 1 (Figure 2a). Most of these occurred by week 8, and the most reported AEs were irritation (*n* = 19), itching (*n* = 17) and tenderness (*n* = 17). Consequently, by week 8, half of MSM required interruption from three-times-per-week dosing (Figure 2b) (Table 2). Reasons for interrupting were exacerbation of pre-existing haemorrhoids (*n* = 3), recurrent anal HSV episodes (*n* = 2), and Grade 2 and above local AEs (*n* = 11). The remaining ten men tolerated the three-times-per-week application continuously for 16 weeks (33%, 95% CI 20.7–54.2); one had Grade 3 pain and redness intermittently and did not require treatment interruption and nine reported mild AEs. These were irritation (*n* = 9), burning (*n* = 6) and itching (*n* = 5). Systemic AEs were present in one participant who reported moderate fatigue at week 8, which resolved following interruptions. Of the 27 men who completed period 1, seven did not want to commit to period 2 due to AEs experienced in period 1, and two men were not compliant with study requirements.

#### 3.1.2. Period 2

Eighteen men agreed to continue into period 2 (66.7%, 95%CI 47.8–81.4). Of note, these men reported less grade 2 AEs in period 1 compared with men who declined period 2 continuation ([55% (10/18) vs. (12.5% 1/9)], *p* = 0.02) During period 2, three men withdrew between weeks 12 and 24 and two between weeks 24 and 36 (Figure 1), leaving 13 men who completed period 2. All men took at least 50% of imiquimod doses and the median number of doses taken over 48 weeks was 43 (IQR 23–48). Kaplan–Meier analysis demonstrated that grade 1 AEs were reported by half of men in period 2; however, grade 2–3 AEs were rare (Figure 2c). The most reported AEs during period 2 were tenderness, itch, irritation and burning on application site. No men required treatment interruption due to AEs during period 2 (Figure 2d) and no systemic AEs were reported.

Two serious AEs were recorded during the study (anal cancer, *n* = 2). Both occurred in period 2. No study-related serious AEs or deaths were recorded.

### 3.2. Human Papillomavirus Genotypes

In period 1, the baseline prevalence of HPV16 and/or 18 was 51.9% (*n* = 14/27, 95% CI 32.0–71.3) (Table 3). Eight men tested positive for HPV16 at baseline and three of these eight men tested negative at week 16 (*p* = 0.35) Seven men tested positive for HPV18 at baseline and three of these seven tested negative at week 16 (*p* = 0.30). The prevalence of HPV16/18 was 33.3% (*n* = 9/27, 95% CI 16.5–54.0) by the end of period 1. The decline in HPV16/8 prevalence was not statistically significant (*p* = 0.08). Infection with more than one hrHPV genotype was common (*n* = 17, 63%). The most frequent genotypes were HPV 39 (*n* = 10, 37.0%), HPV 16 (*n* = 8, 29.6%), HPV 58 (*n* = 8 29.6%), HPV 51 (*n* = 7, 25.9%) and HPV 18 (*n* = 7, 25.9%).

There was no significant association between being HPV16/18 positive at baseline and having moderate to severe AEs (*p* = 0.08).Three men (Study ID 10, 48 and 60) who tested positive for HPV 16/18 in period 1 also completed period 2 (Table 3). One man tested positive throughout period 2, one tested negative at week 48, and one tested negative at week 24 but tested positive again at week 48 (Table 3). Three men who tested positive for hrHPV 16/18 at baseline but tested negative at week 16 (Study ID 23, 37, 42, Table 3) had remained hrHPV negative at weeks 24 and 48 (*n* = 2), and one tested positive again at week 48. Of note, the two men diagnosed with anal cancer during period 2 had hrHPV 16 at baseline, which remained positive in week 16 (Study ID 6 and 35, Table 3).

Overall, we observed that 62% of MSM at week 16 (period 1) and 58% at week 48 (period 2) had decreases in anal HPV viral loads compared to baseline and week 16, respectively (Table 4).

### 3.3. Health-Related Quality of Life and Sexual Functioning of MSM Living with HIV

At baseline, men self-scored their health at a median of 90 points out of 100 (IQR 85–95). No statistically significant changes were observed in the scores during subsequent follow-up points compared to baseline. Health-related quality of life utility weights decreased from a median of 1 at baseline to 0.937 at week 8 (*p* = 0.04) (Supplementary Table S1). Utility weights did not change significantly in the subsequent follow-up points.

Imiquimod use was self-reported to negatively impact the sex life of 28% of MSM at week 8 and 37% at week 16. Reasons were local AEs such as tenderness and the study requirement of not having sex following cream application. In period 2, no men reported their sex life to be impacted by imiquimod use.

## 4. Discussion

In this pilot trial evaluating peri-anal and intra-anal imiquimod cream for anal hrHPV infection, we found three times per week application during period 1 was poorly tolerated and only one-third of men were able to continue the treatment without interruption. Adverse events developed progressively from week 3 and most men required treatment interruption by week 8. In contrast, the men who continued to period 2 had only mild AEs and all tolerated once-a-week treatment. After week 16, some men tested negative for hrHPV 16/18 although new infections were detected in several men at the end of week 48. This study adds to the literature on the tolerability of intra-anal imiquimod in men and it is the first study to evaluate longer-term treatment.

Our results are similar to other studies using imiquimod against established AIN or intra-anal warts. In a study of MSM from the Netherlands of three times a week imiquimod for 16 weeks, almost half of participants reported grade 3–4 AEs (23 men, 43%), and treatment interruption occurred in a quarter of participants (14, 26.4%) [23]. Wieland et al. evaluated a peri-anal regimen of three times per week in 28 men with external AIN and reported local redness and skin erosions in all patients (*n* = 22) as well as poor adherence in 21% [38]. One placebo-controlled study with an efficacy endpoint reported good tolerability over 4 months [39]; however, participants experiencing AEs in this study were instructed not to apply further cream until symptom free and authors did not report the proportion of men who stopped applying the cream due to AEs.

Two men were diagnosed with anal cancer during the study period (Study ID #6 and #55, Table 3). Participant #6 was diagnosed after week 12 of period 2 during routine colonoscopy. This man had HSIL excised 4 years prior and was followed up with annual colonoscopies at a tertiary hospital. He withdrew from the study to commence chemotherapy. Another man was diagnosed after moving interstate and withdrawing from the study before week 24 (Study ID 55 Table 3). No information is available on symptoms, signs or method of anal cancer diagnosis for this participant. No peri-anal or intra-anal lesions were detected while swabbing these men. However, anal cancer screening was not conducted during recruitment. In our HIV clinic, we offer MSM routine screening for anal cancer with a digital anorectal exam at least on an annual basis [7,8] and participants denied having anal cancer in the preceding 12 months as per exclusion criteria. Of note participant #6 showed an increased viral load for all hrHPV genotypes at week 16 (Supplementary Table S2) even though imiquimod therapy is associated with significant decreases in hrHPV DNA loads [40]. Participant #55 had persistent hrHPV 16 and other types at all follow-up points with no viral load changes detected. It should be noted that the signal strength output for the Seegene Anyplex II HPV HR assay is semi-quantitative only, and viral load measurements may vary based on factors in addition to specific template copy number, such as the number of different HPV types found in a sample.

The study had several limitations. First, we did not have a control group and we cannot be sure that changes in HPV types or the presence of anal symptoms would not have occurred without treatment. We cannot attribute the HPV genotype changes to imiquimod without reference to an untreated control group, particularly given HPV infections can self-resolve within two years, and the study was conducted over 16 months [8]. Clinically, however, we do not see grade 2 and above AEs commonly, so it is reasonable to infer that imiquimod was responsible from men developing grade 2 and above AEs. Indeed, we aimed to determine just this; what proportion of men could tolerate three times per week treatment. Second, the exact dose of imiquimod was not precisely measured during the study. Some men reported difficulty ascertaining exactly half a sachet of cream per application, which made consistent dosing challenging. Intra-anal tampons may have been more reliable; however, these were not available in our clinic. Third, the tolerability of once-per-week regimen may be biased towards individuals who experienced less severe AEs with three-times-per-week treatment. Lastly, our study was not powered to detect moderate changes in HPV infection. However, we did not see any large changes in HPV infection during the study, suggesting that if imiquimod were to be effective even at a moderate level it would need to be given for a longer time. Given that once per week was well-tolerated over weeks, future studies should focus on longer durations of treatment and not attempt short time frames. We also noted that once-per-week treatment did not negatively impact on participant’s sex life, further highlighting that this regimen is more sustainable for long-term treatment.

## 5. Conclusions

In summary, self-applied imiquimod three times per week caused treatment-limiting AEs in most men. The tolerability of once per week imiquimod shown in this pilot study suggests that larger studies comparing the effectiveness of imiquimod therapy with placebo to reduce detection of anal hrHPV should be considered.

## Figures and Tables

**Table 1:** Schedule of study follow-up during period 1 and period 2.

	Period 1								Period 2			
Week	0	1	2	3	4	8	12	16 ^a^	12	24	36	48
Anal swab	x							x		x		x
Questionnaires	x					x		x	x	x	x	x
Text message ^b^		x	x	x	x		x		x		x	

^a^ Eligible participants were invited to period 2 enrolment in this visit and those who agreed were. given a 6-month supply of imiquimod. ^b^ In period 1, an automated text message asked participants “Q1: Have you used all three doses of imiquimod in the last 7 days? (if no, how many doses taken) And Q2: Have you had side effects from imiquimod in the last 7 days?”. In period 2, a reminder text message was sent to participants to complete and mail week 12 and week 36 questionnaires. x: Denotes the weeks in which the corresponding follow up (anal swab, questionnaires or text message) was done.

**Table 2:** Adherence to imiquimod by week of follow-up during period 1 and period 2.

Period 1	Week 1	Week 2	Week 3	Week 4	Week 8	Week 12	Week 16
3 doses per week	27 (100.0)	27 (100.0)	25 (92.6)	21 (77.8)	13 (48.2)	14 (51.9)	9 (33.3)
1 dose per week	0 (0.0)	0 (0.0)	1 (3.7)	2 (7.4)	3 (11.1)	9 (33.3)	16 (59.3)
On interruption	0 (0.0)	0 (0.0)	1 (3.7)	4 (14.8)	11 (40.7)	4 (14.8)	2 (7.4)
Men reporting AE	2 (7.4)	7(26.0)	9 (33.3)	11 (40.7)	23 (85.2)	10 (37.0)	22 (81.5)
**Period 2**	**Week 12**	**Week 24**	**Week 36**	**Week 48**			
1 dose per week	18 (100.0)	15 (100.0)	11 ^a^ (100.0)	13 (100.0)			
On interruption	0	0	0	0			
Men reporting AE	7 (39.0)	4 (26.7)	2 (18.2)	2 (15.4)			

Data presented as *n* (%). Abbreviations: AE: Adverse events. ^a^ Two participants did not return the completed questionnaires on week 36, thus we are unable to report their adherence.

**Table 3:** High-risk human papillomavirus types present in the anal samples of men who have sex with men who provided at least two anal swabs.

	Period 1				Period 2			
	Week 0		Week 16		Week 24		Week 48	
ID	16/18	Non-16/18	16/18	Non-16/18	16/18	Non-16/18	16/18	Non-16/18
6 ^a^	16	39, 56, 31	16	39, 56, 31	WD	WD	WD	WD
8	-ve	35, 39, 61, 58, 59	-ve	31, 33, 39, 51, 56, 58, 59	-ve	39, 51, 58, 59	-ve	33, 39, 51, 58, 59, 68
10	18	31, 52, 58	18	33, 58	18	58	-ve	33, 58
14	-ve	45 ^b^	N/A	Sample N/A	WD	WD	WD	WD
16	18	39, 52, 58	-ve	-ve	WD	WD	WD	WD
22 ^a^	-ve	33, 35, 45, 59	-ve	33, 39, 45	-ve	33, 35, 39, 45	-ve	39
23	16	-ve	-ve	-ve	-ve	-ve	-ve	-ve
32	16	-ve	16 ^b^	-ve	WD	WD	WD	WD
34	-ve	39	-ve	39, 66	WD	WD	WD	WD
35	16, 18	33, 51, 58	16 ^b^	51 ^b^	WD	WD	WD	WD
37	16	31, 39	-ve	31, 39	-ve	31, 39	-ve	31, 39
42	18	45, 66, 68	-ve	68	-ve	45, 66, 68	18	45, 56, 66, 68
43 ^a^	-ve	58	-ve	58	-ve	58, 68 ^b^	-ve	58, 68
46 ^a^	16	45	-ve	-ve	WD	WD	WD	WD
47 ^a^	-ve	66	-ve	66	WD	WD	WD	WD
48	18	56, 58	18	56, 58, 68	-ve	56, 68	18	56, 58, 68
49 ^a^	-ve	39, 45, 51, 52, 68	-ve	31, 39, 45, 51, 52, 68 ^b^	-ve	31, 39, 45, 51, 52, 68	-ve	39, 45, 52, 68
53	-ve	51, 56, 58, 68	-ve	51, 56, 58, 68	-ve	51, 56, 68	-ve	51, 58
55	16	31, 39	16	31, 39	WD	WD	WD	WD
58 ^a^	18	45, 59 ^b^	18	51, 59	WD	WD	WD	WD
59	-ve	39	-ve	-ve	WD	WD	WD	WD
60	16	-ve	16	31, 35, 39, 58	16	35, 58	16	31, 35, 39, 58
61 ^a^	-ve	39	-	Invalid sample	-ve	59	16	51, 52, 59, 68
64 ^a^	18	51	16, 18	51	WD	WD	WD	WD
69 ^a^	-ve	35, 51, 58, 56, 68	-ve	33, 56, 58, 68	WD	WD	WD	WD
72	-ve	59 ^b^	-ve	-ve	WD	WD	WD	WD
73	-ve	45, 51, 59	-ve	45, 51, 59	-ve	45, 51, 59	-ve	45, 51, 59

Abbreviations: ID: Study identification number; WD: Withdrawn; -ve: Negative for the corresponding high-risk HPV types; N/A: Not available. ^a^ Denotes patients who were able to tolerate imiquimod cream three times per week continuously in period 1. ^b^ Denotes a sample with invalid internal control result. In these samples, if there was a positive signal for one or more HPV types, we interpreted the negatives in that sample as a true negative and positives as a true positive. In cases where no HPV was detected at all, we interpreted this as an invalid sample.

**Table 4:** Change in human papillomavirus types over period 1 and period 2.

HPV Type Change	Week 16 Compared to Baseline	Week 48 Compared to Week 16
Increased	9/26, 34.6% (19.4–53.8)	3/12, 25.0% (8.9–52.2)
Decreased	16/26, 62.5% (42.5–77.6)	7/12, 58.3 (32.0–80.7)
No change	1/26, 3.9% (0.6–18.9)	2/12, 16.7 (4.7–44.8)

Data presented as *n*/*N*, % (95% CI). Definitions: An increase is either no detection of a specific HPV type followed by detection of that type OR detection at both time points but an increase in the viral load (e.g., + to +++). A decrease is either detection of a specific type at the start but no detection at the end OR detection at both time points but a decrease in the viral load (e.g., +++ to +). No change in HPV type detected at both time points AND the viral load of virus is stable (e.g., ++ to ++).

## Data Availability

Data are contained within the article or supplementary material.

## Supplementary Materials

The following are available online at https://www.mdpi.com/article/10.3390/jcm10194477/s1, Table S1: EQ-5D-5L utility index scores based on participant answers during periods 1 and 2. Table S2: Viral load of high-risk human papillomavirus types present in the anal samples of men who have sex with men who provided at least two anal swabs. Figure S1: Instruction sheet for using imiquimod.
