# Supplementary material for: Intra-Anal Imiquimod Cream against Human Papillomavirus Infection in Men Who Have Sex with Men Living with HIV: A Single-Arm, Open-Label Pilot Study
Source: J Clin Med. 2021 Sep 28;10(19):4477. doi: 10.3390/jcm10194477 (PMC8509144; doi:10.3390/jcm10194477)
Supplement: Supplementary file 1 [file jcm-10-04477-s001.zip › jcm-1359140-supplementary.pdf]

Supplementary Material

Table S1. EQ-5D-5L utility index scores based on participant answers during period 1 and 2

	**Period 1**			**Period 2**			
ID	Baseline	Week 8	Week 16	Week 12	Week 24	Week 36	Week 48
7	0.937	0.937	WD	WD	WD	WD	WD
14	0.922	1	1	WD	WD	WD	WD
16	1	1	1	WD	WD	WD	WD
32	0.809	0.751	0.859	WD	WD	WD	WD
46	1	1	1	WD	WD	WD	WD
47	1	0.937	0.937	WD	WD	WD	WD
55	1	0.673	0.874	WD	WD	WD	WD
58	1	0.887	0.779	WD	WD	WD	WD
59	0.879	0.748	0.937	WD	WD	WD	WD
72	1	1	0.937	WD	WD	WD	WD
6	0.512	0.751	0.481	0.922	WD	WD	WD
34	1	0.937	1	1	WD	WD	WD
64	0.922	1	0.922	0.652	WD	WD	WD
35	0.916	0.937	0.864	1	0.666	WD	WD
69	0.937	0.922	1	1	1	WD	WD
8	1	1	0.859	0.922	0.922	1	1
10	1	1	1	1	1	0.922	0.922
22	1	1	1	0.922	1	WD	1
23	0.922	0.916	0.833	*N*/A	*N*/A	*N*/A	0.775
37	1	0.922	0.937	1	1	0.922	1
42	1	0.717	1	1	1	1	1
43	1	0.922	1	1	1		1
48	1	1	1	1	1	1	1
49	1	1	1	1	1	0.896	1
53	1	1	1	1	1	1	1
60	0.937	0.838	1	0.937	0.937	0.937	0.937
61	1	1	1	1	0.896	1	1
73	0.801	1	0.922	0.922	0.922	0.838	0.922

Abbreviations: ID: Study identification number WD: Withdrawn; N/A: Not available

Table S2. Viral load of high-risk human papillomavirus types present in the anal samples of men who have sex with men who provided at least two anal swabs

	Phase 1 (Intervention)		Phase 2 (Maintenance)	
ID	Week 0<br>HPV type (viral load) [a]	Week 16<br>HPV type (viral load) [a]	Week 24<br>HPV type (viral load) [a]	Week 48<br>HPV type (viral load) [a]
6	16++, 39+, 56+, 31+	16+++, 39+++, 56+++, 31++	WD	WD
8	35++, 39++, 58++, 59++,61++	31++, 33++, 39++, 51++, 56++, 58++, 59++	39+, 51++, 58+, 59+	33++, 39+, 51+, 58++, 59+, 68+
10	18++ 31+, 52+, 58++	18++, 33++, 58+++	18++, 58+++	33+, 58+
14	45+	N/A	WD	WD
16	18+, 39+, 52+, 58++	-ve	WD	WD
22	33++, 35++, 45++, 59+	33++, 39++, 45++	33++, 35+, 39+++, 45++	39+
23	16+++	-ve	-ve	-ve
32	16++	16+	WD	WD
34	39++	39++, 66++	WD	WD
35	16+, 18++. 33++, 51+++, 58++	16+,51+	WD	WD
37	16++, 31++, 39+++	31++, 39++	31+, 39++	31++, 39++
42	18++ 45++, 66+, 68++	68+	45++, 66++, 68++	18++, 45++, 56++, 66++, 68++
43	58++	58++	58++, 68+	58+++, 68+
46	16+, 45++	-ve	WD	WD
47	66++	66++	WD	WD
48	18++, 56++, 58+++	18++ 56++, 58+, 68+	56+, 68++	18+++ 56+++, 58+++, 68+++
49	39++, 45+, 51++, 52++, 68+	31++, 39+++, 45+++, 51+++, 52+++, 68+++	31+, 39+++, 45++, 51++, 52++, 68++	39++, 45++, 52+, 68++
53	51+++, 56++, 58++, 68+	51++, 56++, 58++, 68+	51++, 56+++, 68++	51++, 58+
55	16+++ 31+++, 39++	16++, 31++, 39++	WD	WD
58	18+ 45+, 59+ [b]	18++, 51++, 59+	WD	WD
59	39++	-ve	WD	WD
60	16++	16+++ 31++, 35++, 39++, 58+++	16+, 35+, 58+	16+++ 31++, 35++, 39+++, 58+++
61	39++	Invalid sample	59+	16+, 51++, 52++, 59++, 68++
64	18++, 51+	16+, 18+++ 51+	WD	WD
69	35+, 51+, 58++, 56+, 68++	33++, 56+++, 58++, 68++	WD	WD
72	59++	-ve	WD	WD
73	45++, 51++, 59+	45++, 51++, 59++	45++, 51++, 59++	45+, 51++, 59+

Abbreviations: ID: Study identification number WD: Withdrawn; -ve: Negative for the corresponding high-risk HPV types; N/A: Sample not available

[a] Anyplex assay yields a semi-quantified viral load result which may be high (+++; positive signal before 31 PCR cycles), medium (++; positive signal between 31 and 39 PCR cycles), or low (+; positive signal after 40 PCR cycles)

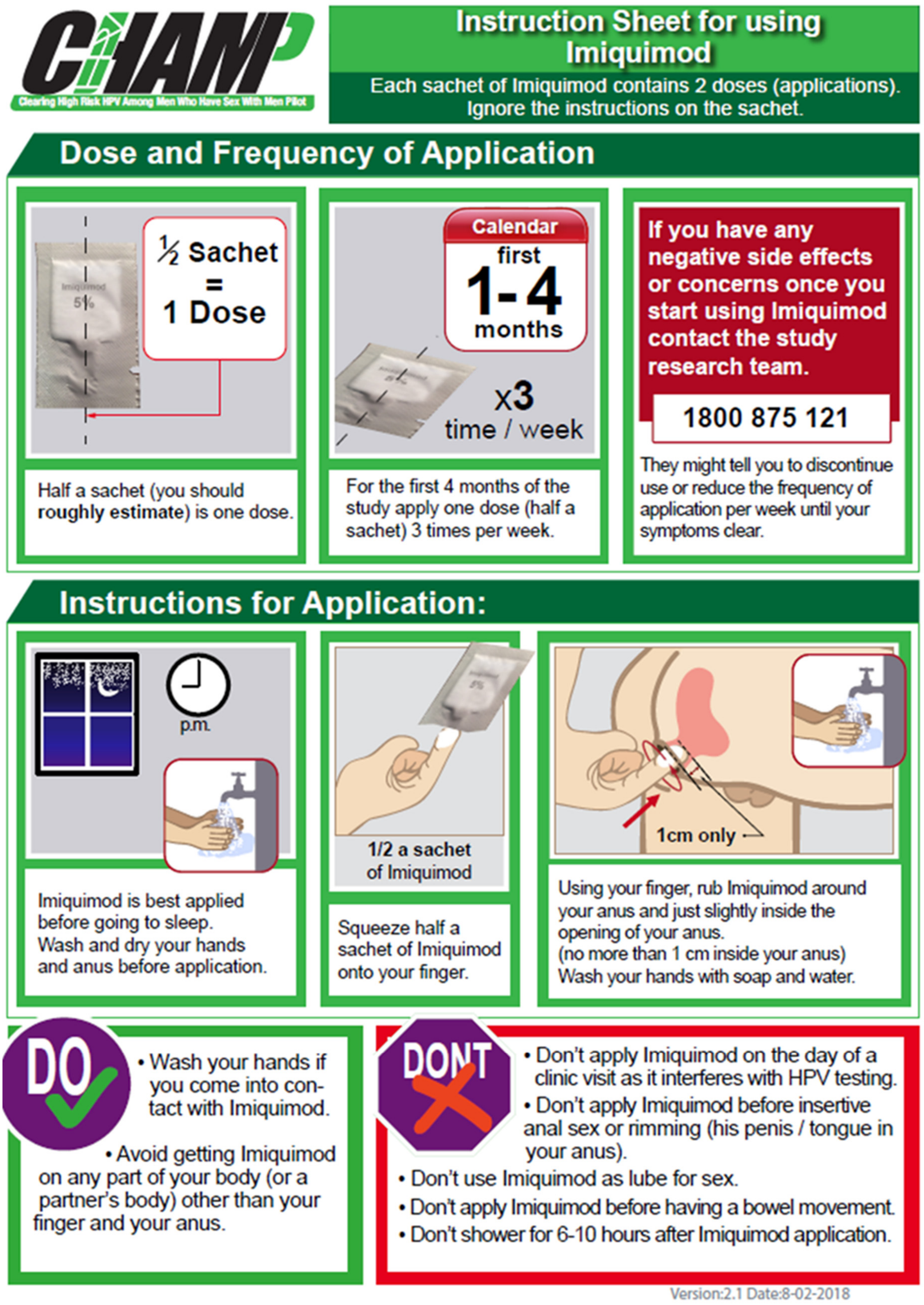

Figure S1. Instruction sheet for using imiquimod.
